# Copeptin Hormone Concentrations in Dogs with Heart Disease and Relationship with Antidiuretic Hormone

**DOI:** 10.3390/ani15071013

**Published:** 2025-04-01

**Authors:** Corine Lavigne, Darcy B. Adin, Courtney Hanner, Alexis Cooper, Rebeca A. Castro, Autumn N. Harris

**Affiliations:** 1 Department of Small Animal Clinical Sciences, College of Veterinary Medicine, University of Florida, 2015 SW 16th Ave, Gainesville, FL 32608, USA; corine.lavigne@ufl.edu (C.L.); adind@ufl.edu (D.B.A.); hannerc0625@ufl.edu (C.H.); cooperalexis@ufl.edu (A.C.); 2 Department of Clinical Sciences, College of Veterinary Medicine, North Carolina State University, 1060 William Moore Dr, Raleigh, NC 27606, USA; rabrugge@ncsu.edu

**Keywords:** canine, cardiovascular, hypochloremia, diuretic resistance

## Abstract

Antidiuretic hormone (ADH) is upregulated in dogs with cardiac disease. However, measurement of ADH is impractical in a clinical setting. Copeptin is co-secreted with ADH, making it a potential biomarker of ADH secretion. We hypothesized that dogs with cardiac disease would upregulate copeptin concentrations and that copeptin would positively correlate with ADH, serum chloride ([Cl^−^]), and degree of mathematical [Cl^−^] correction. The study population comprised 19 healthy, 20 preclinical (Stage B), and 20 congestive heart failure (CHF, Stage C or D) dogs. Groups were compared with Kruskal–Wallis tests. The agreement between ADH and copeptin was assessed with Bland–Altman analysis. Relationships between copeptin and clinical variables were explored using multivariable linear regression. There were no significant differences in copeptin concentrations among healthy (median 54.9 pg/mL; range 0.5–196.1), preclinical (median 43.6 pg/mL; range 0.5–131.4), and CHF (median 60.5 pg/mL; range 0.5–997.8) dogs (*p* = 0.76). Relative to ADH, copeptin showed a negative proportional bias of −87.8 pg/mL with wide limits of agreement (−421.8 to 246.2). Linear regression showed a significant influence of age on copeptin concentration. Copeptin measured by ELISA does not reflect the ADH concentration in healthy dogs or in dogs with cardiac disease. Copeptin concentrations are, however, inversely associated with age.

## Simple Summary

Antidiuretic hormone (ADH) plays a crucial role in regulating water balance in dogs, particularly those with heart disease, where its levels are often elevated. However, measuring ADH in a clinical setting can be challenging. This study explored the potential of copeptin, a substance released alongside ADH, as a more practical biomarker for assessing ADH concentrations in dogs with cardiac issues. We examined 59 dogs divided into three groups: healthy dogs, those in the early stages of heart disease, and dogs with congestive heart failure (CHF). Copeptin concentrations were measured and compared across the groups. Our findings revealed no significant differences in copeptin concentrations among the groups. Additionally, we found that copeptin concentrations did not correlate well with ADH concentrations, suggesting that copeptin may not serve as a direct substitute for measuring ADH with the currently available assays. However, copeptin concentrations were influenced by age after controlling for potential explanatory variables, with an inverse relationship. Overall, while lower copeptin concentrations were associated with increasing age, copeptin does not effectively reflect ADH concentrations in dogs with cardiac disease, highlighting the need for further research in this area.

## 1. Introduction

Patients with congestive heart failure (CHF) have chronic sympathetic nervous system and renin–angiotensin–aldosterone system (RAAS) stimulation, which leads to vasoconstriction and sodium and water retention [1,2,3]. Medications used to treat CHF affect diuresis, prevent fluid retention, and improve forward flow. Despite these therapies, some dogs become refractory in the advanced stages of CHF and continue to retain fluid, a state that is marked by poor diuretic responsiveness and low serum electrolyte concentrations [4,5,6,7,8,9]. Free water retention results in hyponatremia, hypochloremia, and low serum osmolarity because of non-osmotic release of antidiuretic hormone (ADH, also known as vasopressin) from the pituitary in response to poor forward flow and angiotensin II stimulation [3,10,11]. In a healthy state, ADH is released in response to changes in osmolarity. For instance, in patients with dehydration, increased serum osmolarity will stimulate ADH release to restore blood volume [10]. Studies in people with CHF demonstrated high ADH concentrations despite low plasma osmolarity in some patients [3,11,12]. Similarly, dogs with CHF have increased ADH concentrations [3,13,14].

Hypochloremia and hyponatremia in CHF from free water retention due to non-osmotic release of ADH have been associated with a poor prognosis [8,15,16,17]. Mathematically corrected [Cl^−^] (c[Cl^−^]) using the following equation: c[Cl^−^] = (mid-reference range sodium concentration [Na^+^] ÷ measured [Na^+^]) × measured [Cl^−^], which normalizes the chloride concentration to the sodium concentration, can be used to assess the dilutional impact of water on serum [Cl^−^] in patients with heart disease [18]. A positive difference between measured and corrected [Cl^−^] indicates water retention. Hypochloremia due to renal loss from loop diuretic effects results in a lack of notable change after mathematical correction. A mixed effect of dilution and depletion on serum [Cl^−^] concentration occurs in some dogs with CHF [18]. We have previously shown that dogs with advanced CHF that are poorly responsive to standard therapies (stage D) have a larger gap between measured and corrected [Cl^−^] concentrations than dogs with controlled CHF (stage C) [6]. Mathematically correcting [Cl^−^] might provide insights into water balance.

Previous research from our group has shown that ADH is upregulated in dogs with cardiac disease compared to healthy dogs [19]. This increase in ADH release is suspected to occur secondary to non-osmotic factors such as decreased cardiac output and activation of the RAAS, leading to relatively excessive free water retention. Dogs with elevated ADH concentrations might benefit from using ADH antagonists or RAAS antagonists to prevent cardiac disease progression and improve outcomes. However, the measurement of ADH is impractical in part due to the hormone’s short half-life and instability. 

Copeptin, the C-terminal segment of the arginine vasopressin precursor peptide, is co-secreted with ADH and has shown promise as a surrogate biomarker for ADH in people [20,21,22]. Copeptin has been investigated as a marker of fluid balance and thermal strain potentially related to tonicity changes during heat stress and dehydration states where osmolality is increased [23]. For similar pathophysiologic reasons, copeptin appears useful in people to diagnose syndromes of inappropriate ADH and psychogenic polydipsia because it is an indicator of body fluid homeostasis [24]. Furthermore, copeptin predicts outcomes in people with advanced heart failure [25,26,27,28]. Therefore, regardless of stimuli being tonicity related or uncoupled to osmolality as in CHF states, copeptin might be a clinically applicable biomarker in dogs with heart disease that could be useful in identifying dogs with excessive ADH secretion that could benefit from additional therapy aimed at decreasing free water retention or antagonizing the underlying RAAS stimulation.

The primary objective of this study was to determine if serum copeptin concentrations are related to the canine heart disease stage. We also sought to assess the agreement of copeptin and ADH and to explore associations between serum copeptin and indirect biomarkers of RAAS activation and water retention, including ADH, [Cl^−^], serum osmolality, and degree of mathematical [Cl^−^] correction in dogs with cardiac disease. We hypothesized that dogs with heart disease would have higher copeptin concentrations than healthy dogs and that copeptin would show good agreement with ADH.

## 2. Materials and Methods

This study was performed using banked serum samples from a previous prospective study at the University of Florida, Small Animal Hospital [19]. Healthy dogs were recruited from students, faculty, and staff, while dogs with heart disease were seen through the cardiology service. Informed client consent was obtained from all owners. The Institutional Animal Care and Use Committee at the University of Florida approved blood collection for this study (202120518).

### 2.1. Animals

Stored serum samples collected from dogs with heart disease and healthy control dogs enrolled in a previously study were used for this study [19]. Inclusion criteria were the absence of clinically significant extra-cardiac disease, no administered medications other than loop diuretics that have the potential to affect copeptin or ADH concentrations (e.g., acetazolamide, nonsteroidal anti-inflammatory medications, corticosteroids, opioids), and sufficient residual stored serum. Dogs were separated into groups based on the presence or absence of and severity of heart disease using published guidelines for heart disease staging, where dogs were Stage B if they had heart disease without clinical signs (Stage B1 without cardiomegaly and Stage B2 with cardiomegaly), Stage C if they had CHF controlled with medications, and Stage D if they had refractory CHF [2]. Dogs were fasted the morning of the appointment, and clinical variables were recorded, including signalment, body weight, sex, and heart disease stage.

### 2.2. Electrolyte Measurements and Calculated Osmolality

Whole blood (1.3 mL) was obtained via peripheral venipuncture, and serum was used for the measurement of renal panels (blood urea nitrogen, creatinine, sodium (Na^+^), potassium, Cl^−^, and bicarbonate), which were performed using an AU480 Chemistry Analyzer (Beckman Coulter, Brea, CA, USA) through the Clinical Pathology Laboratory at the University of Florida as previously reported [19]. Serum electrolytes (Na^+^, potassium, Cl^−^) and laboratory-reported calculated serum osmolality (calOsm = 2[Na^+^] + [glucose/18] + [blood urea nitrogen/2.8] were recorded for each dog. The measured serum [Cl^−^] was mathematically corrected to obtain the corrected serum [Cl^−^] (c[Cl^−^]) using the following equation: (mid-reference range [Na^+^]/measured [Na^+^]) × measured [Cl^−^]. The amount of [Cl^−^] correction was determined by subtracting the measured [Cl^−^] from the c[Cl^−^].

### 2.3. Assay Measurements

Protease-inhibited plasma was used for the measurement of ADH concentrations using a commercially available, previously validated enzyme immunoassay following the protocol recommended by the kit manufacturer (Arbor Assay, Ann Arbor, MI, USA, Accessed on 27 September 2024, https://www.arborassays.com/product/arg8-vasopressin-avp-elisa-kit/), the details of which have been previously reported for these samples [14,19]. The lower limit of detection was 2.11 pg/mL for the ADH assay.

Serum samples from the same venipuncture that were used to obtain plasma for ADH measurements were stored for less than 6 months at −80 °C and used for measurement of copeptin concentrations with a commercially available enzyme immunoassay following the protocol recommended by the kit manufacturer (MyBioSource, Inc., San Diego, CA, USA, Accessed on 27 September 2024, https://www.mybiosource.com/cpp-canine-elisa-kits/copeptin/736743). The lower limit of detection was 1.0 pg/mL for the copeptin assay.

### 2.4. Statistical Analysis

The data were compiled in an Excel spreadsheet (Microsoft Office, Redmond, WA, USA) and statistically analyzed using the commercially available software Graph Pad Prism (GraphPad Software, Boston, MA, USA, version 9). Measured values for ADH and copeptin that were at the lower limit of detection were recorded as half the value for statistical analysis. Descriptive and bloodwork data for dogs in each group were tested for normality using the Kolmogorov–Smirnov test and presented as the median and range (minimum–maximum) due to several variables showing a non-normal distribution, including the primary variable of interest (copeptin). Stage C and D CHF dogs were combined into one Stage C/D group. Age, weight, [Na^+^], [Cl^−^], corrected [Cl^−^], amount of [Cl^−^] correction, ADH, and copeptin concentrations were compared among healthy dogs, Stage B dogs, and Stage C/D dogs using Kruskal–Wallis tests or one-way ANOVA, depending on the data distribution, with Dunn’s test or the Holm–Šídák’s test, respectively, performed for multiple comparison testing. Fisher’s exact test was used to compare sex among groups. The agreement between ADH and copeptin concentrations was assessed by Bland–Altman analysis, in which ADH was used as the reference. Bias was defined as the mean difference between the two measurements, and the 95% limit of agreement (LOA) was calculated as the bias ± (1.96 × SD). Multivariable linear regression was used to assess the influence of potential explanatory variables that could be associated with serum copeptin concentrations. Variables were selected based on a biologically plausible effect or those identified as different among groups with *p* < 0.1. Variables with high collinearity were not included and the model was evaluated for goodness of fit. This model included age, serum [Cl^−^], calOsm, use of RAAS inhibitors (e.g., angiotensin-converting enzyme inhibitors, spironolactone), loop diuretic dosage (expressed as furosemide equivalency where torsemide dose × 15 was considered equivalent to furosemide dose) [29] and ADH concentrations. Significance was set at *p* < 0.05.

## 3. Results

Nineteen healthy dogs, 20 preclinical (Stage B) dogs, and 20 CHF (Stage C/D) dogs met the inclusion criteria with a sufficient volume of stored serum between 18 September 2023, and 19 October 2023 [19]. Stage B dogs included 13 Stage B1 and seven Stage B2 dogs. Dogs with CHF included 16 Stage C and four Stage D dogs. Of the dogs with heart disease, 36 had degenerative mitral valve disease, three had DCM, and one had mitral valve dysplasia. Clinical details of the included dogs have been previously reported [19].

**Figure 1:**
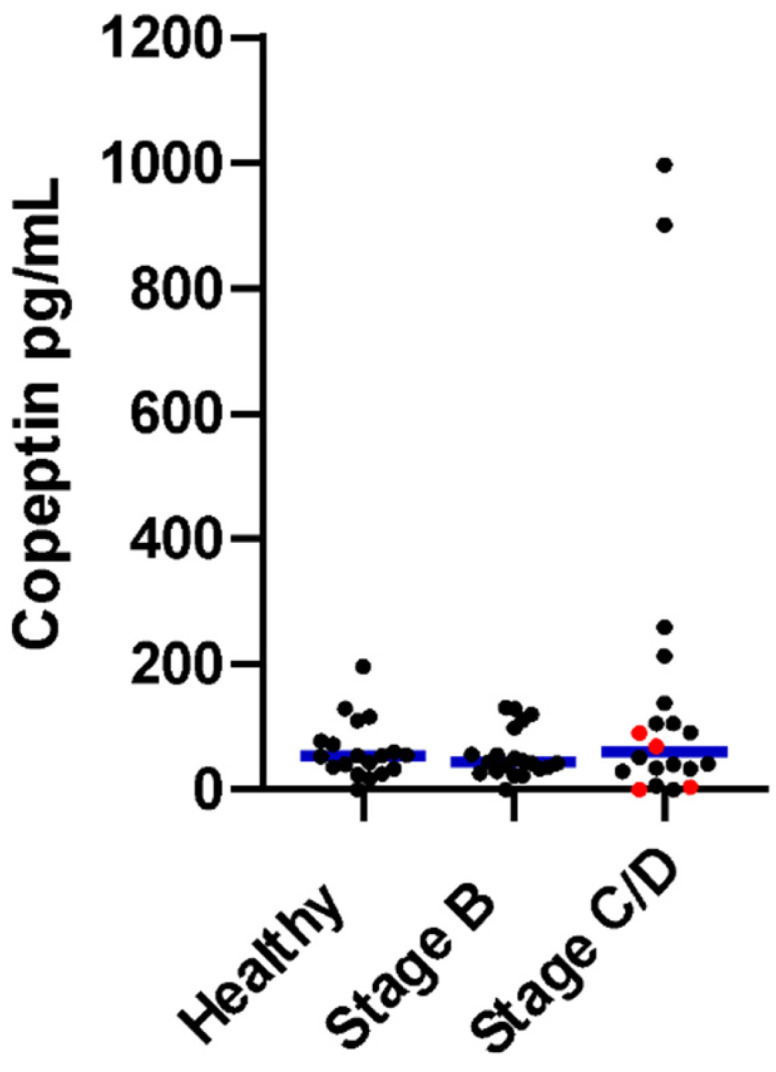
Copeptin hormone concentrations in healthy dogs, preclinical (Stage B) heart disease dogs, and congestive heart failure (Stage C/D) dogs. Stage D dogs are depicted by the red dots. The blue horizontal line represents the median value for each group. There was no significant difference between groups (*p* = 0.76).

Table 1 shows the comparison of values for each group. The copeptin concentration did not differ among healthy, Stage B, and Stage C/D dogs (Table 1 and Figure 1). Serum [Cl^−^] and corrected [Cl^−^] significantly differed among all three groups, with Stage C/D (CHF) dogs having the lowest and healthy dogs having the highest values. No group differences were detected for the amount of [Cl^−^] correction or [Na^+^] (Table 1). The ADH concentration was higher in preclinical (Stage B) and CHF (Stage C/D) dogs compared to healthy dogs, but there was no difference between preclinical (Stage B) and CHF (Stage C/D) dogs, as previously reported (Table 1) [19].

Multivariable linear regression analysis showed that age was significantly and inversely associated with serum copeptin concentration, while the other explanatory variables ([Cl^−^], calOsm, ADH, loop diuretic dosage, and the use of RAAS inhibitors) were not associated with copeptin (Table 2). The model passed normality with low collinearity and moderate goodness of fit (adjusted R^2^ = 0.5244).

**Figure 2:**
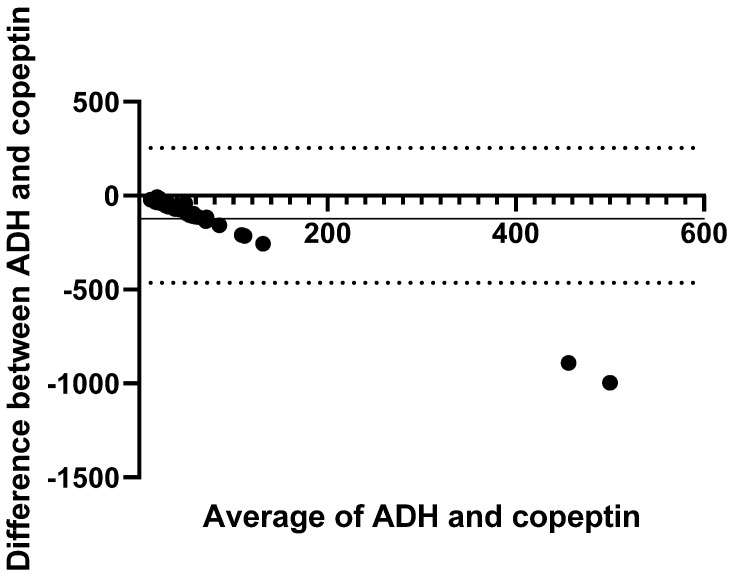
Bland–Altman plots for analysis of the agreement between ADH and copeptin concentrations of all dogs (n = 49). The solid horizontal line represents the bias, and the dotted lines represent the 95% limit of agreement. ADH (antidiuretic hormone) concentrations were used as the reference.

Relative to ADH, copeptin showed poor agreement, with a negative proportional bias of −87.8 pg/mL and wide limits of agreement (−421.8 pg/mL to 246.2 pg/mL) (Figure 2).

## 4. Discussion

The major finding of this study is the lack of a significant relationship between ADH and copeptin concentrations as hypothesized. This was shown by poor method agreement between these two variables that should both be measures of pro-vasopressin release. This finding contrasts with studies in people in which copeptin has been shown to be closely related to ADH over a wide range of plasma osmolality [30]. Both copeptin and ADH peptides are thought to be stimulated by similar physiological processes, such as osmotic stimulation, hypovolemia, or stress [31,32,33]. However, while the physiological function of ADH is homeostasis of fluid balance, vascular tonus, and regulation of the endocrine stress response, the physiological function of copeptin is much less clear [21,31,33]. Copeptin is postulated to be a chaperone-like molecule that is involved in the formation of pro-vasopressin and is thought to respond as rapidly as ADH to osmotic, hemodynamic, and unspecified stress-related stimuli, thereby resulting in equimolar production together with ADH [21,30,32]. Since much less is known about the regulation and function of copeptin, there could be differences between dogs and humans in the regulation and function of copeptin, leading to the lack of an association between ADH and copeptin in the dogs in this study.

Furthermore, different immunoassays exist to measure copeptin. The two assays used most often in human clinical studies are the original sandwich immune luminometric assay (LIA) and its automated immunofluorescent successor [34,35]. In addition, various enzyme-linked immunosorbent assays (ELISAs) have been developed [36,37]. A recent study in people showed that copeptin measured by the LIA or immunofluorescent assay showed a good correlation over a wide range of copeptin concentrations [38]. In contrast, copeptin measured by ELISA had a poor correlation with the other two assays and had poor diagnostic accuracy [38]. This poor correlation between the ELISA and the other two types of copeptin assays could be due to the detection of other epitopes on copeptin with the antibodies used, different rates of copeptin fragment formation, other substances being measured with the ELISA assay, or heterophile antibody production. It is possible that the measurement of canine copeptin concentrations with an LIA or immunofluorescent assay would show a better correlation between ADH and copeptin concentrations. Unfortunately, those types of assays are not currently available for the measurement of canine copeptin. Therefore, based on the results from this study, copeptin measured via ELISA should not be used as a surrogate marker for ADH concentrations.

The second major finding from this study was a lack of a difference in copeptin concentrations between healthy dogs and dogs with heart disease (Stages B or C/D), which was in contrast to what was hypothesized. Given the lack of a significant association between copeptin and ADH concentrations found in this study, it is unsurprising that there were no significant group differences between healthy dogs and dogs with heart disease. This was unexpected based on what has been shown in a recent meta-analysis of human studies, which concluded that elevated copeptin concentrations are associated with all-cause mortality in patients with heart failure [39]. Furthermore, in humans with acute myocardial infarction, copeptin concentrations were higher in patients who died or were re-admitted with heart failure compared with survivors [26]. In a multivariate analysis in people, copeptin was an independent predictor for death or heart failure [26,27,28]. However, our study design differed from those performed in people in that we looked at group differences and agreement, but not outcomes. Based on the studies in people, future research endeavors might consider assessing the predictive utility of copeptin concentrations for survival in dogs.

The final major finding from this study was the significant inverse association of age with copeptin concentration that was found using multivariable linear regression. The reason for the inverse relationship between copeptin and age is currently unclear, and further evaluation is needed. No correlation between age and copeptin concentration was found in healthy human subjects, which is in contrast to findings in people with kidney disease and children with heart failure, which showed a positive correlation between copeptin and age [40,41,42]. The response axes for copeptin release might be less effective in older animals, as can occur with other physiologic feedback mechanisms [43]; however, this is a postulation without supportive evidence in the literature.

Copeptin does not appear to be related to ADH in dogs based on our study results, but it is possible that the release stimulus is separate from both physiologic osmoregulation and non-osmotic ADH release in heart disease [44]. Since copeptin concentration has been shown to be associated with all-cause mortality and is an independent predictor of death and heart failure in humans, further analysis of the eventual outcome of the dogs in this study might inform the use of copeptin for this purpose [26,27,28,39]. The significance of the influence of age on copeptin in an inverse manner, however, is unexpected and requires further study.

Limitations of this study should be considered during the interpretation of our results. Temporal and biological variability of copeptin and ADH have not been reported. Since study samples were collected during clinical appointments, differences in the time of collection or fasting state might have impacted the results. Even though copeptin is reported to be more stable than ADH and all samples in this study that were used for copeptin measurement were stored at −80 °C for less than 6 months before the measurements were performed, there might have been variability in the results related to degradation or loss of sample through processing or storage.

## 5. Conclusions

The results of this study demonstrated poor agreement between copeptin and ADH concentrations, indicating that copeptin measured by the currently available ELISA assay should not be used as a surrogate marker for ADH. The influence of age on the copeptin concentration requires further study.

## Figures and Tables

**Table 1:** Demographic and bloodwork values from healthy dogs, dogs with preclinical (Stage B) heart disease, and dogs with congestive heart failure (Stage C/D). Data represent the median and range.

Variable	Healthy	Preclinical (Stage B)	CHF (Stage C/D)	*p* Value
Age (months)	62 (24–148)	124 (82–191) *	144 (37–182) *	**<0.001**
Weight (kg)	15.7 (3.4–32.6)	8.6 (3.2–32.9)	8.6 (4.2–71.5)	0.10
Sex	0 FI, 11 FS, 0 MI, 8 MC	0 FI, 8 FS, 1 MI, 11 MC	0 FI, 8 FS, 3 MI, 9 MC	0.46
[Na^+^] (reference range 141.9–150.6 mEq/L)	146.4 (141.1–148.6)	146.8 (145.1–151.6)	145.4 (138.7–153.7)	0.19
[Cl^−^] (reference range 107.8–117.1 mEq/L)	113.8 (109.4–117.6) #	111.3 (105.4–115.7) *	108.0 (97.9–112.5) *#	**<0.001**
Corrected [Cl^−^] (mEq/L)	113.7 (109.4–117.6) #	110.6 (104.4–113.8) *	107.3 (100.1–113.9) *#	**<0.001**
Amount of [Cl^−^] correction (mEq/L)	−0.10 (−1.9–4.1)	−0.40 (−4.1–0.9)	0.70 (−5.4–5.6)	0.17
Antidiuretic hormone (pg/mL)	3.4 (0.1–6.2)	6.5 (1.8–33.8)*	6.7 (2.0–28.1) *	**0.007**
Copeptin hormone (pg/mL)	54.9 (0.5–196.1)	43.6 (0.5–131.4)	60.5 (0.5–997.8)	0.76

* significantly different from healthy dogs, # significantly different from preclinical (Stage B) dogs (*p* < 0.05 considered statistically significant). CHF: congestive heart failure; [Cl^−^]: chloride concentration; FI: female intact; FS: female spayed; MC: male castrated; MI: male intact; [Na^+^], sodium concentration.

**Table 2:** Multivariable linear regression model to identify predictors of serum copeptin concentrations. The *p* value for the model was 0.011. β is the beta coefficient and indicates the directionality of the relationship.

Variable	β (95% CI)	Adjusted *p*
Age (months)	−4.52 (−7.12 to −1.92)	0.002
Loop diuretic dosage	−19.40 (−49.55 to 10.76)	0.19
[Cl^−^]	−6.31 (−41.84 to 29.22)	0.71
calOsm	−3.65 (−16.37 to 9.06)	0.55
ADH	−9.07 (−14.55 to 32.69)	0.42
RAAS inhibitor use	−158.6 (−407.8 to 90.51)	0.19

ADH: antidiuretic hormone; calOsm: calculated osmolality; [Cl^−^]: chloride concentration; RAAS: renin–angiotensin aldosterone system.

## Data Availability

Data are contained within the article.

## Abbreviations

ADH	antidiuretic hormone
ACVIM	American College of Veterinary Internal Medicine
calOsm	calculated serum osmolality
Cl^−^	chloride
CHF	congestive heart failure
cCl^−^	corrected serum chloride concentration
DCM	dilated cardiomyopathy
LOA	limit of agreement
RAAS	renin-angiotensin aldosterone system
SD	standard deviation
Na^+^	sodium
